# Pulpal and Periodontal Tissues Changes Associated with Le Fort I and Sagittal Split Ramus Osteotomies: A review

**DOI:** 10.2174/1874210601812010024

**Published:** 2018-01-29

**Authors:** Konstantinos Lazaridis, Maria Lazaridou, Athanasios E. Athanasiou

**Affiliations:** 1 Department of Orthodontics, Faculty of Dentistry, Aristotle University of Thessaloniki, Thessaloniki, Greece; 2 Department of Oral and Maxillofacial Surgery, Faculty of Dentistry, Aristotle University of Thessaloniki, Thessaloniki, Greece; 3 Department of Orthodontics, Hamdan Bin Mohammed College of Dental Medicine, Mohammed Bin Rashid University of Medicine and Health Sciences, Dubai, United Arab Emirates

**Keywords:** Le Fort I osteotomy, Sagittal split osteotomy, Orthognathic surgery, Pulpal blood flow, Tooth discoloration, Maxilla

## Abstract

**Introduction:**

Le Fort I and sagittal split ramus osteotomies are the most commonly performed orthognathic surgery procedures on the maxilla and mandible, respectively.

**Techniques:**

Despite progress in the techniques, these procedures may still be associated with morbidity, expressed as inflammation, inadequate bony union, periodontal damages or in extreme cases even total bone loss.

**Discussion:**

Through a comprehensive review of the literature, the influences of maxillary and mandibular surgery on Pulpal Blood Flow (PBF), pulp sensitivity and pulp vitality are examined. Moreover, adverse effects of maxillary surgery on tooth color and periodontal tissues are also reported. The effects had a variety of expression. Concerning maxillary surgery, some studies showed an initial increase in PBF followed by a decrease to the baseline or even lower levels after 1-3 months. Other studies found an initial decrease in PBF followed by an increase soon after. There were also studies that showed no significant PBF changes, in contrast.

**Conclusion:**

Concerning mandibular surgery, a recent study showed a decrease in PBF immediately after sagittal split ramus osteotomy. Some authors detected tooth discoloration of maxillary teeth after Le Fort I osteotomy. Root resorption and root injury were also detected, but were of minor significance. Usually, these adverse effects derive from injury of the vessels of the palatal pedicle. This pedicle should be maintained intact for the avoidance of blood flow impairments. In addition, the descending palatine artery should be protected during maxillary surgery procedures in order to maintain the highest possible blood flow on the maxillary teeth.

## 1 INTRODUCTION

Le Fort I osteotomy is the most commonly applied osteotomy on the maxilla, whereas the sagittal split ramus osteotomy remains the procedure of choice on the mandible. Despite accumulated excessive experience, morbidity may be associated with these surgical procedures including inflammation [1], inadequate bony union [2], periodontal damages [3, 4], and in extreme cases total loss of the osteotomized bone [5, 6]. Through a comprehensive review of the literature, this article addresses possible adverse effects of these surgical procedures on pulp and periodontal tissues as well as the methods applied to measure their severity. Recommendations to minimize them will also be discussed with emphasis on the preservation of adequate blood supply by the avoidance of injury of important vessels.

The literature review was conducted using PubMed/Medline, Google Scholar and EMBASE by two independent reviewers. Articles were searched with the following terms: “blood-flow and Le Fort I osteotomy,” “blood-flow and orthognathic surgery,” “vascular impairments and orthognathic surgery,” “effects of Le Fort I osteotomy,” and “effects of orthognathic surgery.”. Abstracts and their original articles were selected for review from the available titles. Disagreements were resolved by discussion and third-party review. The initial search was performed on January 14^th^, 2016. All articles with reports of vascular impairments or any other effects on teeth and periodontal tissues related to Le Fort I osteotomy were comprehensively reviewed. Bibliographies of included articles were subsequently reviewed for additional publications.

## 2 INFLUENCES OF MAXILLARY SURGERY ON PULPAL BLOOD FLOW

Vascular impairment is considered as the main factor for the development of severe pulpal damages following orthognathic surgery procedures. Vitality tests give little information on blood flow because the innervation of the pulp may be compromised, specifically on patients that have undergone maxillary surgery [7].

Ramsay **et al.** (1991) used a Periflux PF3 laser flowmeter (Perimed, Stockholm, Sweden) in order to record blood flow at specific time points before and after Le Fort I surgery. A randomly selected right or left mandibular canine was used as a control. The findings revealed the presence of an initial ischemic phase. Despite the variability of the blood flow measurements after this ischemic phase, it was shown that a phase of hyperemia occurred afterwards. According to the authors, these blood flow changes can affect the pulp although no total interruption of blood flow was recorded. A clinical finding of this study was the reduction of periodontal support on one patient with discoloration of one central incisor. Despite the discoloration and the loss of periodontal support, no significant difference in the blood flow was found on this particular tooth [8].

Geylikman **et al.** (1995) using Laser Doppler Flowmetry (LDF) examined a group of 12 consecutive patients receiving Le Fort I osteotomy with control group surgical patients who received mandibular surgery and one non-surgical untreated group. The authors measured Pulpal Blood Flow (PBF) of the right and left maxillary incisors and Gingival Blood Flow (GBF) from a site slight apical to the interdental papilla. The time intervals were (a) before surgery, (b) 0-8h after surgery, (c) 8-16h after surgery, and (d) 16-24h after surgery. Their results revealed no significant change in pulpal blood flow, but in contrast showed a significant drop in gingival blood flow in the Le Fort I group. Especially on a patient who experienced severe bone and gingival loss after surgery, the decrease of gingival flow was remarkable [9].

Buckley **et al.** (1999) performed a study to evaluate the use of LDF in the measurement of PBF following orthognathic surgery and the effects of Le Fort I osteotomy on the blood supply of the maxillary central incisors. The study group (group I) consisted of 15 subjects receiving single Le Fort I advancement osteotomy. The first control group (group II) included 7 single mandibular advancement osteotomy patients, while the second control group (group III) was a non-invasion group of 20 patients. The measurements were performed utilizing two methods of flowmeter positioning. The first was a handheld supporting method and the second was with the application of a stabilizing splint. There was no consistent difference or significant random variation between the two methods. Their results showed no significant PBF differences on the control groups, however on the maxillary study group they demonstrated an initial rise in the PBF of the maxillary central incisors followed by an overall reduction in comparison with the pre-surgical reading at 6 months [10].

In a similarly designed study Justus *et al*. (2001) demonstrated an initial increase in PBF of the maxillary central incisors on the Le Fort I study group, which was not present on the two control groups. PBF was recorded in maxillary incisors and GBF was assessed from a site slightly apical to the interdental papilla of the maxillary central incisors in 10 patients who were undergoing a Le Fort I osteotomy (study group), 10 patients who were undergoing a mandibular osteotomy (control group I), and 10 nonsurgical control subjects (control group II) who were not undergoing orthodontic treatment. Blood flow measurements were made before surgery and at intervals between 7 and 10 days, 14 and 17 days and finally between 21 and 24 days after surgery. Analysis of the results showed an increase in PBF between the first and third week after Le Fort I osteotomy, but no significant change in GBF [11].

Emshoff *et al*. (2000) studied 12 subjects undergoing segmented Le Fort I osteotomy without the use of a control group. Using LDF they investigated PBF on maxillary incisors, canines and premolars on three time-points: immediately before surgery, 4 days after surgery and 56 days after surgery. They found a significant decrease in PBF of lateral incisors, canines and first premolars in short-term and long-term measurements. They concluded that teeth adjacent to the vertical cuts of segmented osteotomies are those mostly affected by surgery [12].

At the same clinic (Emshoff *et al.*, 2008) a redesigned study investigated PBF on patients undergoing segmented Le Fort I osteotomy (study group I) and patients undergoing non-segmented Le Fort I osteotomy (study group II). As control group, subjects undergoing no surgery and no orthodontic treatment were used. Measurements were taken before surgery (session I), 3-5 days after surgery (session II) and 55-59 days after surgery (session III). They found a tooth-related statistically significant reduction of PBF in the region of maxillary canines on the segmental osteotomy group, occurring between session I and II. According to the authors this confirms the concept that teeth adjacent to the osteotomy cuts may be affected by ischemia during the early phase after surgery. They also studied the possibility of appearance of adverse outcomes such as the presence of PBF reduction >40%. A significant increase in risk of a session II related adverse outcome occurred with a non-segmented and segmented Le Fort I osteotomy. The odds ratio that a patient with segmented Le Fort-I osteotomy might belong to the session III related adverse outcome group was strong. All the above indicate that Le Fort-I osteotomy is an important prognostic determinant of adverse PBF outcomes [13].

In an effort to increase the data on tooth specific PBF changes, Eroglu and Sabuncuoglu (2014) designed a study where data were acquired from maxillary incisors, canines and first premolars. The study group consisted of 14 Le Fort I patients, while 7 patients with mandibular surgery formed the first control group and 7 non-invasion patients formed the second control group. The measurements took place at the baseline (before surgery), at week 1 after surgery and at months 1, 3, 6 and 12 after surgery. The longitudinal LDF readings from all 28 patients in a period of 12 months revealed a PBF reduction in all tooth types after Le-Fort I osteotomy. The effect of Le Fort I osteotomy varied by tooth type. Dramatic reductions of PBF were recorded on some patients shortly after the operation specifically on canines and first premolars. Hyperemia was observed in lateral incisors between months 1 and 3 postoperatively. No absence of PBF was recorded at any time. PBF gradually but significantly recovered over time, although never reached the preoperative baseline levels [14].

Dodson *et al*. (1996) raised the question whether the decreased GBF observed during the intraoperative course of Le Fort I osteotomy is due to the operation or due to the vasoconstriction caused by the use of local anesthetic. They used a randomized clinical trial design by assigning patients in a Le Fort I surgery group including local anesthetic (19 patients) and a second Le Fort I group (15 patients) without local anesthetic. The main study variable was GBF while other study variables included age, sex, additional lower jaw osteotomy, blood loss, osteotomy movement magnitude, temperature, pulse, O_2_ saturation, and surgical time. The mean GBF decreased more rapidly in the first group in the beginning of the operation while by an average of 2.3 hours into the operation the mean GBF was equivalent in both study groups. They concluded that the use of local anesthetic significantly affects GBF during the early phase of the operation, but the effect dissipates in the time period between soft tissue dissection and maxillary down fracture [15].

Harada **et al.** (2003) examined the change in blood flow and recovery of sensibility in the maxillary dental pulp during and after maxillary distraction. Their study group included 5 patients undergoing high Le Fort I osteotomy and maxillary distraction and their control group 14 patients who underwent a common single-segment Le Fort I osteotomy and mandibular setback surgery. Eleven maxillary incisors of the study group and 54 maxillary incisors of the control group were assessed preoperatively and at 1-7 days, 14 days, and 3 months postoperatively. PBF was measured by LDF. From postoperative days 1 to 5 (the latency period), the PBF tended to be higher in the study than in the control group. From day 6 to 3 months postoperatively (during and after maxillary distraction), the PBF values of the 2 groups were similar. Maxillary distraction is shown to be a favorable technique for maintenance of PBF in the early phase [16].

At the same clinic (Harada *et al*, 2004) a comparison took place regarding PBF changes between single-segment Le Fort I osteotomy (group I) and Le Fort I and horseshoe osteotomy (group II). Group I included 14 patients, while group II included 9 patients. Thirty-two maxillary incisors from the first group and 54 maxillary incisors from the second group were examined preoperatively and at 1-7 days, 14 days, and 3, 6, and 12 months postoperatively. The pulpal blood flow was measured by LDF. In both groups, the PBF dropped to its lowest value on day 1 after surgery and increased thereafter. A temporary drop of the PBF was observed in the single segment osteotomy group on day 4, which was not present on the horseshoe osteotomy group. Their results suggest that both maxillary osteotomy methods influence the postoperative PBF [17].

The findings of the above-mentioned investigations are presented in summary in Table **1**.

3

INFLUENCES OF MANDIBULAR SURGERY ON PULPAL BLOOD FLOW

With the use of LDF, Chen *et al*. (2011) reported a decrease in PBF after Sagittal Split Ramus Osteotomy (SSRO), which started to recover after 18-28 weeks, although never reached preoperative levels. In patients where SSRO was accompanied with genioplasty, there was a constant, although not statistically significant, higher drop of blood flow during the first two weeks followed by a higher recovery rate. This recovery started as a trend notable after 4-6 weeks and reached a peek at weeks 10-16. Despite the tendency to recover, postsurgical blood flow never reached the levels of pre-surgical measurements similarly with the non-genioplasty group [18].

Von Arx *et al*. (2007) investigated the pulp sensitivity and vitality of mandibular incisors and canines before and after bone harvesting in the region of symphysis. They examined 20 patients that were addressed for symphysis bone grafting procedure. Pulp vitality tests were performed with carbon dioxide, while LDF was used for the estimation of blood flow. The mandibular incisors and canines were evaluated preoperatively, postoperatively and 6 months after surgery with the two methods. The results showed that teeth with sensitivity changes demonstrated the greatest decrease (a statistically significant decrease) of PBF over time. Loss of pulp sensitivity appeared to be correlated to a significant decrease of blood flow assessed by LDF [19].

The findings of the above-mentioned investigations are presented in summary in Table **2**.

## 4 INFLUENCES OF MAXILLARY AND MANDIBULAR SURGERY ON PULP SENSITIVITY AND VITALITY

Sato **et al.** (2003) examined blood flow change and recovery of pulp sensibility of the maxillary incisors in patients undergoing a single segment Le Fort I osteotomy. They measured PBF preoperatively and then at day 1 to day 7, 2 weeks, 3 months, 6 months and 1 year after surgery. Pulp sensibility was simultaneously recorded on the same time intervals. The authors found that the mean PBF dropped to its lowest value at day 1 after surgery, then started to recover except from day 4 where a slight drop was observed. All teeth showed negative pulp sensibility from day 1 to day 14 after surgery. Sensibility gradually started to recover and by the third month half of the teeth were positive. The authors showed that the teeth with recovered sensibility on the third month had significantly higher PBF by day 1 in comparison to the teeth that still had negative sensibility by the third month. The authors concluded that the decrease in PBF that is associated with single-segment Le Fort I osteotomy, may affect pulp sensibility in the short term after surgery [20].

Harada **et al.** (2004), in addition to PBF tests, performed pulp vitality tests by means of electrodiagnostics to compare the effects of different types of maxillary osteotomies. They found that pulpal sensibility recovered easier in 14 patients with single-segment Le Fort I osteotomy, than in 9 patients with a combination of Le Fort I and horseshoe type osteotomy. In order to perform the comparison, 32 incisors of group I and 54 incisors from group II, where addressed for pulp sensibility tests. Furthermore, the same authors compared pulp sensibility of a five patient group who were addressed for maxillary distraction with a 14 patient group who were addressed for a common single segment osteotomy. The results showed that immediately after surgery both groups were negative to pulp sensibility tests, but in a three-month control period the distraction group showed a much higher recovery rate. Numerically 90.9% of teeth in the distraction group were positive in the pulp test, whereas only 50.0% of teeth of the single-segment group showed positive response. As a conclusion, single-segment Le Fort I osteotomy is more favorable in comparison to segmented techniques, but distraction techniques are even more favorable than single-segment maxillary operation in terms of recovery of teeth sensibility [17].

Mesgarzadeh *et al*. (2010) conducted a comprehensive study on the effects of Le Fort I osteotomy on the maxillary anterior teeth. The sample included 32 women and 10 men who were investigated for Le Fort I osteotomy. Postoperatively, the anterior teeth of the upper jaw were examined for pulp vitality using electric pulp tester (EPT), cold tests, heat tests, percussion and palpation. Each test was performed 3 times in intervals of 30 seconds, 60 seconds and 120 seconds respectively. The mandibular anterior teeth served as controls. The cold test showed positive responses on 91% of patients, the EPT on 88.8% of patients, while the heat test on 89.4% of patients, all in a control period from 1 to 5 years after the operation. Only a 3.2% of teeth needed a root canal treatment [21].

## 5 INFLUENCES OF MAXILLARY SURGERY ON TOOTH COLOR

Buckley *et al*. (1999) claimed that Le Fort I osteotomy impairs the maxillary blood supply and results in noticeably decreased viability accompanied by discoloration of the front maxillary teeth [10]. Mesgarzadeh *et al*. (2010) found a discoloration rate of 5,3% of their patients [21]. There was no significant relationship between pulp vitality and discoloration. Thus discoloration associated with Le Fort I osteotomy is not necessarily associated with pulp necrosis. In addition to the initial ischemia that was followed by hyperemia in the study group of Ramsay **et al.** (1991), gray discoloration of one maxillary incisor was also discovered. This was the only discoloration in a group of 14 surgical patients. It is of high importance to mention that to this particular patient the left great palatal artery was severed during the operation [8].

## 6 INFLUENCES OF MAXILLARY SURGERY ON PERIODONTAL TISSUES

Mordenfeld and Andersson (1991) evaluated periodontal and pulp condition on 20 patients who underwent transverse expansion of the maxilla by combined midline and Le Fort I osteotomy. They performed tooth percussion sound test, tooth mobility measurements, pulp sensibility tests, pocket depth measurements, alveolar bone level measurements and radiographic estimation of possible root resorption or root injury. All teeth of all patients showed normal (dull) percussion and physiologic mobility rate. The mean pocket depth in the mesial and distal sulci of the central incisors was 2.2 mm and 2.1 mm respectively with no statistically significant differences. The mean marginal bone levels were 3.1 mm mesial and 2.7 mm distal to the central incisors. These differences reached statistical significance. In one tooth resorption was detected on the mesial root surface. Minor root surface injury, on the apical third, was detected on two central incisors. It is expected that it derived from the saw during the operation. Another tooth showed a small area of root resorption. The authors concluded that although there might be some minor effects on pulp and periodontium, the clinical significance seems negligible [22].

Carroll **et al.** (1992) compared 20 orthodontic patients with equal number of patients receiving combined orthodontic-surgical treatment. The periodontal status was evaluated for a period from 1 to 10 years after surgery. The parameters investigated were plaque index, gingival index, tooth mobility, width of keratinized tissue, probing depth, gingival recession and attachment level. No statistical difference was found between the two groups. Patients with maxillary osteotomy were subdivided in patients receiving total Le Fort I osteotomy and patients receiving segmented osteotomy. No significant differences were found for patients with osteotomies segmentized between the central incisors. However, a statistically significant increase in probe depth and loss of attachment level of up to 0.3 mm was found at the sites of osteotomies segmentized between the canine and second premolar. The authors described this difference as clinically irrelevant [3].

Ueki *et al*. (2006) proposed an original approach of orthodontic management in segmental maxillary osteotomy. They engaged elastic power chain just after surgery on square sectioned orthodontic wires. This allowed for a quick formation of periodontal papilla in the segmentation site between canines and premolars. By comparing these patients with patients who received standard procedures they found that patients of their original method were not prawn to the development of gingival, marginal or other periodontal defects. Moreover, the rate of alveolar bone increased on these patients and decreased on the control patients. They suggested that their technique may prevent periodontal issues that usually occur at the interdental osteotomy site [23].

## 7 DISCUSSION

Before the introduction of LDF, blood flow was only measurable by means of radioactive isotopes or by hydrogen gas clearance. Both methods posed stress as the former requires the injection of the radioactive substance and the latter the insertion of an electrode into the blood vessel. LDF is a noninvasive method firstly introduced by Yeh and Cummins (1964) to measure blood flow in capillaries [24]. When LDF was applied to measure pulpal and gingival blood flow after orthognathic surgery, vascular changes were detected. These effects had a variety of expression. For instance concerning Le Fort I osteotomy, some studies showed an initial increase in PBF followed by a decrease to the baseline or even lower levels after 1-3 months [10, 11]. On the contrary, other studies found an initial decrease in PBF followed by an increase soon after [8]. There are also studies that showed no significant PBF changes in contrast to the previous authors [9]. Concerning mandibular surgery, it has been shown that a definite decrease in PBF occurs immediately after sagittal split ramus osteotomy. Despite recovery, PBF never reached pre-surgical levels [18]. It has been also found that reduction of PBF also occurs during bone harvesting in symphysis region [19]. Although the PBF measurements showed a variety of results, it is definite that in both Le Fort I and sagittal split ramus osteotomies a change of PBF does occur. The same influence could be detected during localized surgery in the symphysis region. If Le Fort I osteotomy is performed correctly, there is usually no risk of permanent injury to the blood supply of the maxillary teeth. However, if a transient reversible vascular impairment does happen, or if the tooth apices are severed during the osteotomy pathologic changes within the pulp may occur.

The occurrence of blood flow changes related to the osteotomies may cause degenerative and atrophic pulpal changes. Animal studies revealed fibrosis and loss of odontoblast layer in the early postsurgical period [25], whereas re-innervation was detected after a 6-month postsurgical follow up [26]. In addition, Yoshida *et al.* (1996) found regressive pulpal changes immediately after surgery, whereas the number of pulp cells was shown to increase substantially in the late stage compared with that in early stage after single tooth dento-osseous osteotomy [27].

According to Epker (1984a) there may be six basic difficulties in the effort to elucidate the vascular effects of the orthognathic surgery. These are failure to describe the specifics of the surgical technique that is applied, failure to mobilize and reposition the surgical segments, inherent errors in the techniques of investigating vascular supply, failure to correlate quantitative histology of the bones with quantitative vascular changes, improper time interval after surgery and finally failure to evaluate the spectra of vascular compromise sequelae [5]. With the sagittal ramus osteotomy of the mandible, ischemic sequelae were considered quite rare. Despite this belief the recent work of Chen *et al.* (2012) demonstrated a reduction in PBF in patients undergoing this osteotomy type, posing further consideration [18]. On the other hand, the possibility of the occurrence of ischemic adverse effects is much higher in maxillary surgery. The primary blood supply to the buccal alveolus, periodontium and teeth derives from the posterior superior alveolar vessels. The primary supply to the palate and palatal alveolus derives from the greater palatine vessels, whereas the buccolabially attached gingiva and adjacent free mucosa are primarily supplied *via* the underlying bone and not *vice versa* [28]. This anatomic fact renders very important the preservation of the palatal pedicle, which should not be transected or crimped within osteotomized segments. The blood supply of the buccal pedicle would be in that case inadequate and adverse necrotic effects would be expected. Segmental Le Fort I osteotomy with simultaneous transverse expansion is not a favorable procedure to ensure blood supply. Instead a total subapical osteotomy is preferable in such cases because it protects the palatal pedicle and reduces blood supply issues [6]. A horse-shoe type osteotomy which preserves the descending palatine arteries is preferable to standard Le Fort I osteotomy because it minimizes the possibility for ischemic adverse effects [17]. Discoloration of teeth was evident in cases where the greater palatine artery was severed rather than in cases that these vessels remained intact [8].

## CONCLUSION

Maxillary surgery may have adverse effects including pulpal blood flow changes, pulp sensibility issues and in some cases tooth discoloration, which is not necessarily connected to pulp necrosis. The segmented Le Fort I osteotomy increases the risk for blood supply impairments especially on teeth immediately adjacent to interdental osteotomy sites. Usually, the adverse effects derive from injury of the vessels of the palatal pedicle. This pedicle should be maintained intact for the avoidance of blood flow impairments. Moreover, the descending palatine artery should be protected during maxillary surgery procedures in order to maintain the highest possible PBF and GBF to maxillary teeth. Although no vascular impairments were expected in the past from a sagittal split ramus osteotomy of the mandible, a reduction of PBF was also shown on this surgical approach with the use of LDF. Further investigation and qualitative surgical procedures are needed for the minimization of these cases.

## Figures and Tables

**Table 1:** Effects of Le Fort I osteotomy on Pulpal Blood Flow (PBF) and Gingival Blood Flow (GBF) studied by means of Laser Doppler Flowmetry (LDF).

**Publications**	**Findings on PBF and GBF Using LDF**
Ramsay *et al.* (1991)	Initial ↓ in PBF (ischemia) followed by ↑ in PBF (hyperemia)
Geylikman *et al.* (1995)	no significant change in PBF, significant ↓ in GBF
Buckley *et al.* (1999)	Initial ↑ in PBF immediately after surgery, followed by ↓ 6 months after surgery in comparison to presurgical levels
Justus *et al.* (2001)	↑ in PBF at weeks 1-3, no change in GBF
Emshoff *et al.* (2000)	↓ in PBF
Emshoff *et al.* (2008)	↓ in PBF
Harada *et al.* (2004)	↓ PBF in single segment Le Fort I compared to distraction osteotomy
Harada *et al.* (2004)	↓ PBF of longer duration (4days after surgery) in single segment osteotomy vs ↓ PBF (1 day after surgery) in horseshoe type osteotomy
Eroglu and Sabuncuoglu (2014)	↓ PBF short after the operation, ↑ PBF 1-3 months after the operation

**Table 2:** Effects of mandibular surgery on Pulpal Blood Flow (PBF) studied by means of Laser Doppler Flowmetry (LDF).

**Publications**	**Findings on PBF using LDF**
Chen *et al.* (2011)	↓ In PBF in SSRO patients, postsurgical PBF never reached presurgical levels ↓ In PBF (remarkable higher) in SSRO+ genioplasty patients
Von Arx *et al.* (2007)	↓ In PBF during bone harvesting in symphysis region
